# Establishment of HeLa Cell Mutants Deficient in Sphingolipid-Related Genes Using TALENs

**DOI:** 10.1371/journal.pone.0088124

**Published:** 2014-02-03

**Authors:** Toshiyuki Yamaji, Kentaro Hanada

**Affiliations:** Department of Biochemistry and Cell Biology, National Institute of Infectious Diseases, Shinjuku-ku, Tokyo, Japan; University of Nantes, France

## Abstract

Sphingolipids are essential components in eukaryotes and have various cellular functions. Recent developments in genome-editing technologies have facilitated gene disruption in various organisms and cell lines. We here show the disruption of various sphingolipid metabolic genes in human cervical carcinoma HeLa cells by using transcription activator-like effector nucleases (TALENs). A TALEN pair targeting the human *CERT* gene (alternative name *COL4A3BP*) encoding a ceramide transport protein induced a loss-of-function phenotype in more than 60% of HeLa cells even though the cell line has a pseudo-triploid karyotype. We have isolated several loss-of-function mutant clones for *CERT*, *UGCG* (encoding glucosylceramide synthase), and *B4GalT5* (encoding the major lactosylceramide synthase), and also a *CERT/UGCG* double-deficient clone. Characterization of these clones supported previous proposals that CERT primarily contributes to the synthesis of SM but not GlcCer, and that B4GalT5 is the major LacCer synthase. These newly established sphingolipid-deficient HeLa cell mutants together with our previously established stable transfectants provide a ‘sphingolipid-modified HeLa cell panel,’ which will be useful to elucidate the functions of various sphingolipid species against essentially the same genomic background.

## Introduction

**Figure 1:**
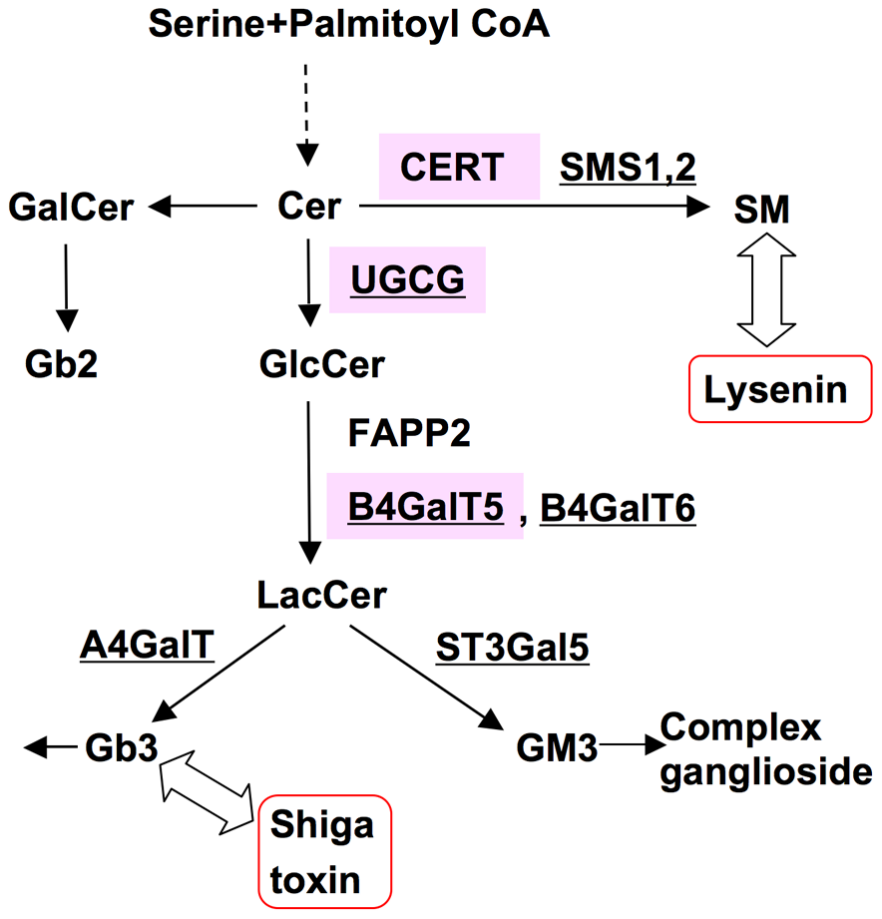
Sphingolipid biosynthesis in mammalian cells and sphingolipid binding toxins. The biosynthetic pathway of sphingolipids relevant to this study is shown. Underlining indicates enzymes for sphingolipid biosynthesis. Pink-shaded boxes indicate the products of genes that were targeted by TALENs in this study. Red-lined boxes indicate the toxins used in this study. Cer, ceramide; SM, sphingomyelin; GlcCer, glucosylceramide; LacCer, lactosylceramide; Gb3, globotriaosylceramide; GalCer, galactosylceramide; Gb2, galabiosylceramide (Galα1-4GalCer); SMS, sphingomyelin synthase; UGCG; UDP-glucose: ceramide glucosyltransferase, B4GalT5, β-1,4-galactosyltransferase 5; B4GalT6, β-1,4-galactosyltransferase 6; FAPP2, four-phosphate adaptor protein 2.

Sphingolipids are essential components of eukaryotes [1]–[3]. In mammalian cells, sphingolipids play important roles in various biological events, including proliferation, apoptosis, differentiation, and adhesion [4]–[9]. Besides their physiological roles, sphingolipids are also involved in the pathogenesis of several diseases, and alteration of sphingolipid metabolism affects diabetes [10]–[12], neuronal diseases including Alzheimer's disease [13], [14], and infectious diseases [15]. Ceramide is the key intermediate for the biosynthesis of sphingomyelin (SM) and glycolipids, which are the major sphingolipids in the plasma membrane (Figure 1). *De novo* biosynthesis of ceramide occurs at the cytosolic surface of the endoplasmic reticulum (ER), and the synthesized ceramide is transported to the Golgi apparatus where SM and glucosylceramide (GlcCer) are synthesized. The ER-to-Golgi trafficking of ceramide includes two pathways, vesicular trafficking and non-vesicular trafficking [16]–[19]. The ceramide transport protein CERT mediates ER-to-Golgi non-vesicular trafficking of ceramide, which is required for the synthesis of SM but not GlcCer [16]. CERT contains two organelle-targeting regions, a pleckstrin homology (PH) domain bound to the Golgi and a short peptide motif designated FFAT bound to the ER, and these bindings permit efficient and directional trafficking of ceramide [16], [20]. GlcCer is synthesized by UDP-glucose:ceramide glucosyltransferase (gene symbol *UGCG*, encoding GlcCer synthase), which transfers glucose (from UDP-glucose) to ceramide mainly at the cytosolic face of the Golgi apparatus [21]–[23]. Then, GlcCer is converted to lactosylceramide (LacCer) on the lumen side of the Golgi apparatus [24]. Two enzymes, β-1,4-galactosyltransferase 5 and 6 (B4GalT5 and 6), are known to function as LacCer syntheases, and analyses of knockout mice showed that B4GalT5 rather than B4GalT6 is the major LacCer synthase [25]–[27]. Gene-targeted mice deficient in *CERT*, *UGCG*, and *B4GalT5* showed embryonic lethality, which indicates the physiological importance of these genes [28]–[30].

Since being established from a biopsy of cervical carcinoma in a female in 1951 [31], the HeLa cell lineage has been greatly contributed to many research fields of life sciences [32]. HeLa cells can continuously proliferate with a relatively short generation time and are applicable to various culture conditions, compared to other mammalian cultured cell types. HeLa cells are susceptible to various microbes, such as poliovirus and the obligate intracellular parasitic bacteria *Chlamydia trachomatis*, making the cells to be a good host cell model of microbe infection [33], [34]. Conventional methods of somatic cell genetics, including chemical mutagenesis, cell colony isolation, DNA transfection, and RNA interference (RNAi), are well applicable to HeLa cells. Moreover, the haplotype genome and epigenome of HeLa cells have recently been resolved [35], [36].

We have studied the metabolism and functions of sphingolipids in mammalian cultured cells [37], [38]. Recently, we found that the transmembrane BAX inhibitor motif containing family proteins may affect subcellular localization of a trans-Golgi enzyme responsible for the synthesis of the glycosphingolipid Gb3, which serves as the membrane receptor of Shiga toxin, after isolation and analysis of HeLa cell variants resistant to Shiga toxin [39]. To further elucidate the sphingolipid biology, we assumed that collecting HeLa cell variants defective in specific sphingolipid-related genes with essentially the same genomic background would be useful.

The RNAi method has been widely used to repress gene expression in cultured cell lines because of its widely applicable convenience. However, since RNAi does not necessarily repress the expression of its target gene perfectly, it is sometimes insufficient to induce loss-of-function of the target gene. Such insufficiency often occurs in the case of partial reduction of enzymes, because enzymes act as catalysts in the biochemical metabolism and, thus, partial reduction of enzymes may not lead to the supposed reduction of their metabolites. This problem would be overcome if the gene of the target enzyme were disrupted by recently developed genome-editing technologies, including transcription activator-like effector nucleases (TALENs) [40].

Each TALEN monomer consists of a TAL effector containing customizable DNA binding repeats and the catalytic domain of FokI endonuclease. Since the FokI domain functions as a dimer, a pair of TALENs is designed to recognize the target genome with proper spacing, where a DNA double-strand break (DSB) is introduced specifically. DSBs are often repaired by non-homologous end-joining (NHEJ), which results in insertions or deletions (indels). Consequently, two-thirds of the repairs cause a frameshift, most of which lead to translational termination and loss of function. Several reports have shown the improvement of genome-editing efficiency in TALENs as well as zinc finger nucleases, another genome-editing nuclease, by structural modifications of the nucleases [41]–[44], and transient hypothermia after transfection of TALEN pairs [41], [45].

In this study, we succeeded in creating loss-of-function mutants for several sphingolipid metabolic genes, including *CERT*, *UGCG*, and *B4GalT5*, in a HeLa cell line by TALEN technology. A panel of these HeLa cell mutants will facilitate sphingolipid biology in human cultured cells.

## Materials and Methods

### Cell Culture, Antibodies, and Reagents

HeLa-mCAT#8 cells, which express mouse cationic amino acid transporter 1 (serves as the mouse ecotropic retroviral receptor) [39], [46], were maintained in Dulbecco's modified Eagle's medium (DMEM) containing 10% fetal bovine serum (FBS). Peroxidase-conjugated rat anti-HA IgG (3F10) and 3-(4,5-dimethylthiazoyl-2-yl)-2,5-diphenyltetrazolium bromide (MTT) were from Roche Diagnostics. Mouse anti-α tubulin IgG (DM1A) and lysenin were from Sigma. Rabbit anti-COL4A3BP (CERT) antibody was from Abcam (Cambridge, UK). Puromycin was from Nacalai Tesque (Kyoto, Japan). Shiga toxin 1 (Stx1) and Alexa-555 Stx1 B subunit (555-Stx1B) were described previously [39]. Thin-layer chromatography (TLC) plates (Silica Gel 60) and High-performance TLC (HPTLC) plates (Silica Gel 60) were from Merck. l-[U-^14^C]serine (5.957 GBq/mmol) was from Moravek (Brea, CA), and d-[1-^14^C]galactose (2.072 GBq/mmol) was from GE Healthcare. All primers used for PCR are described in Supporting Information.

### Synthesis of TALEN constructs

**Figure 2:**
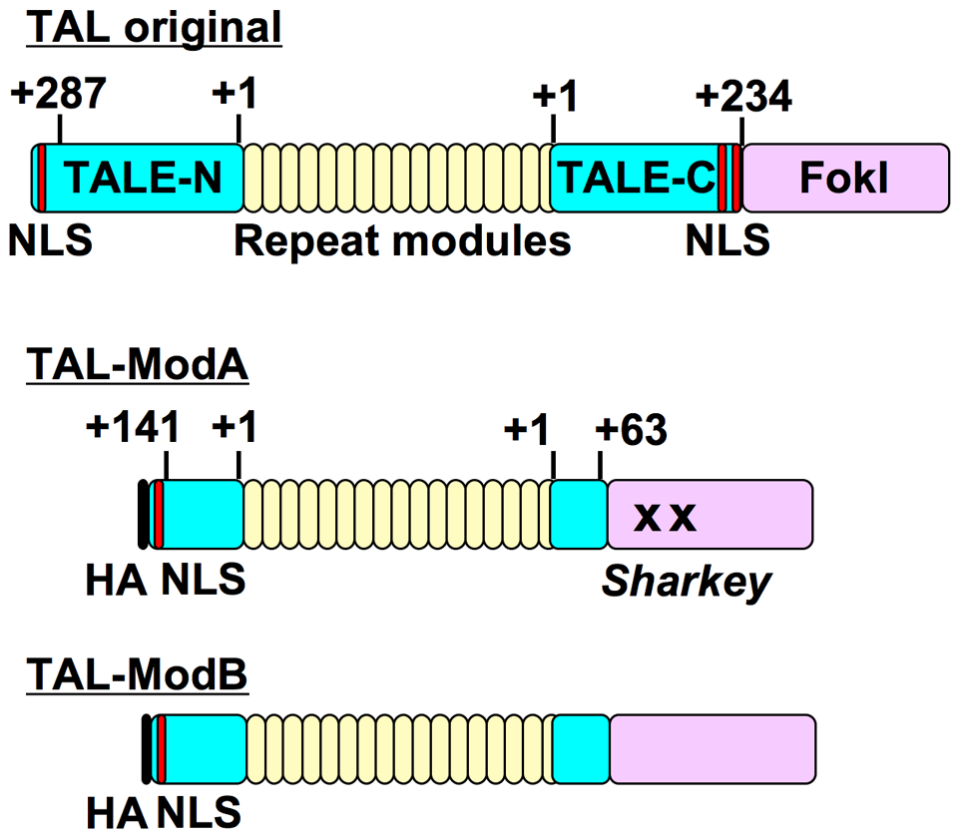
Modified TALEN structures. A, Original and modified TALEN structures. TAL-A and TAL-B contain truncated N-terminal (+141) and C-terminal (+63) TALE domains (ΔNΔC), and TAL-A also contains a mutated FokI, *Sharkey*, instead of the wild type. TALE-N, N-terminal TALE domain; TALE-C, C-terminal TALE domain; HA, hemagglutinin tag; NLS, nuclear localization signal. The amino acid sequences of these TALEN scaffolds and their numbering are shown in Figure S1.

The original “Golden Gate TALEN and TAL Effector Kit”, developed by the Voytas group, was obtained through Addgene (Cambridge, MA) [47]. Modified pTAL plasmids, pTAL-ModA and -ModB, were constructed from the original pTAL3 using a PCR-based mutagenesis kit (PrimeStar GXL or PrimeStar Max; Takara Bio, Otsu, Japan) and a seamless cloning method (GeneArt Seamless Cloning and Assembly; Life Technologies). Primers used for modifying the original plasmid and the amino acid sequences of the modified TALEN scaffolds are described in Supporting Information and Figure S1. As shown in Figure 2, both pTAL-ModA and -ModB contain a truncated TALE scaffold (N+141/C+63) with an HA tag and a nuclear localization signal at the N-terminal of the TALE scaffold (DDBJ/EMBL/GenBank accession number; AB872042 and AB872043), which was designed based on the previous paper [41]. pTAL-ModA further contains a mutated FokI, *Sharkey*, instead of the wild type (DDBJ/EMBL/GenBank accession number; AB872044) [44]. Both plasmids are compatible with the Golden Gate assembly. All TALENs were designed using TALE-NT or TALE-NT 2.0 web-based tools [47], [48], and the used repeat variable di-residues (RVDs) are shown in Figure S2. TALEN module assembly was performed according to the manual and the original report [47]. After a second Golden Gate reaction, pTAL3 or the modified pTAL vectors containing full-length TALEN were cleaved with XhoI and AflII, and the insert was ligated into pSELECT-puro-mcs with a modification at the multicloning site (pSELECT-puro-L1). The size of pSELECT-puro is only about 3.4 kbp in spite of the expression vector, and the puromycin-resistant gene cassette is used for both *E. coli* and mammalian cells. Expression of a TALEN gene is driven by the hEF1-HTLV promoter.

### Construction of retroviral vectors and retroviral infection

Construction of pMXs-IP–hB4GalT5-HA vector was as follows: human *B4GalT5* cDNA was amplified by PCR (template; brain cDNA (Invitrogen), primers; B4GalT5 RI-ATG and B4GalT5 HindIII-END) (DDBJ/EMBL/GenBank Accession number; AB871482). The amplified DNA was digested with EcoRI and HindIII, and inserted into pCXN2-cHA [39]. pCXN2–hB4GalT5-HA was digested with EcoRI and NotI, and the fragment was inserted into pMXs-IP. pMXs-IP–hB4GalT6-HA vector was constructed previously [39]. The preparation of retrovirus particles and their infection of HeLa-mCAT#8 cells were performed as described previously [39], [49].

### Transfection of TALEN constructs

(Day 0) HeLa-mCAT8 cells (1.5×10^5^ cells/well in 12-well plates) were cultured overnight. (Day 1) A pair of TALEN plasmids was mixed with X-tremeGENE HP (Roche Diagnostics) (in 12-well plates, 0.5 µg each of plasmids and 2 µl X-tremeGENE HP were mixed in 100 µl Opti-MEM), and then the mixture was added to the cells. (Day 2) The cells were transferred to 6-well plates and cultured at 37°C with puromycin at 5 µg/ml, which is higher than the usual concentration to concentrate cells with a higher expression of TALENs. (Day 3) The plates were moved to 30°C. (Day 4) The medium was changed to puromycin-free medium and the cells were kept at 30°C. (Day 7) The cells were subcultured and grown at 37°C for a few days. The TALEN-treated HeLa cells were harvested for indel analysis and lysenin treatment, or diluted to isolate gene-disrupted clones.

### Indel analysis

Genomic DNA was isolated with a Blood Genomic DNA Extraction Mini Kit (Favorgen, Ping-Tung, Taiwan). In some cases, trypsinized cells were simply heated in TE buffer followed by vortexing to use as a template of genomic PCR. PCR was performed with PrimeSTAR GXL, and then blunt-end PCR products were cloned with a Zero Blunt TOPO PCR Cloning Kit (Invitrogen). After *E.coli* transformation, colony direct PCR or plasmid purification was performed to use as a template of sequence analysis. DNA sequences were determined with an ABI3100 Genetic Analyzer (Applied Biosystems). Description of the predicted proteins was based on the recommended mutation nomenclature [50]. For example, p.Thr61GlnfsTer30 means a frame shifting change (fs) with Thr97 as the first affected amino acid, changing into a Gln, and the new reading frame ending in a stop (Ter) at position 30. p.Asp59_Thr61delinsAla means a substitution of Asp59–Thr61 for Ala (delins). p.0? means the case that no protein production is expected due to a change in the translation initiation codon.

### mRNA analysis

RNA isolation and reverse transcription were performed as described previously [39]. PCR, cloning, and DNA sequences were performed as described above.

### Lysenin and Stx treatment

**Figure 3:**
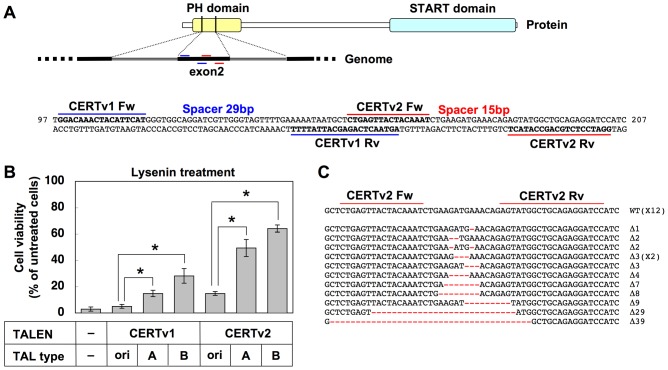
Evaluation of *CERT* gene disruption by TALEN-CERT pairs in HeLa cells. A, Target sites of TALENs-CERT pairs (version 1 and 2) in human *CERT* gene. The sequences are located in exon 2, which codes part of the PH domain. The target sites are shown in bold. The numbers on the right and left of the sequence indicate the sequence numbers from the A of the translation initiation codon, based on *CERT* mRNA (accession number AY4533859). B, Resistance to lysenin in TALEN-CERT–treated HeLa cells. Six TALEN-CERT pairs, which contain two pairs of repeat modules directed against different target sites (CERTv1 and v2) contained in three TALEN scaffolds (TAL original (ori), TAL-ModA, and TAL-ModB), as well as the empty vector (–). These TALEN pairs were transfected into HeLa-mCAT#8 cells, followed by 30°C incubation. The cells were treated with lysenin at 800 ng/ml for 2 h. Their viability was estimated by the MTT assay and is expressing as a percentage of the value (OD570) in the absence of lysenin: mean percentage ± S.D. obtained from three independently repeated experiments. The Bonferroni corrected *t*-test was used for multiple comparisons. *, *p*<0.017. C, Indel analysis of TALEN-CERT–treated HeLa cells. The pair of target sites is shown in bold. Indels are shown in red and their lengths are specified on the right of the sequences.

The cytolytic activity of lysenin (Sigma) was examined first. HeLa-mCAT#8 cells (5×10^4^ cells/well in 24-well plates) were cultured overnight, and treated with lysenin at various concentrations (200–1600 ng/ml) for 1 h. MTT assay was performed as described previously [39]. The minimum concentration of lysenin to kill the cells completely was 400 ng/ml (data not shown), and we used 650–800 ng/ml in this study. The concentration of Stx1 used in this study was 100 pg/ml and Stx1 treatment was performed as described previously [39]. All experiments, including the transfection of various TALEN-CERT pairs in Fig. 3B, were repeated three times independently and viability was expressed as the mean percentage ± S.D. obtained from three independent experiments. Student's *t*-test with Bonferroni correction was used for statistical analysis, setting p<0.017 (0.05 divided by 3) as a statistical significance criterion.

### Metabolic labeling of sphingolipids and TLC analysis

Cells (2–3×10^5^ cells) were seeded in a 6-well plate. After overnight culture in the normal culture medium, the medium was changed to Opti-MEM with 1% Neutridoma-SP (Roche) and the cells were incubated with 18.5 kBq of l-[U-^14^C]serine or 6.1 kBq of d-[1-^14^C]galactose for 16 h. Extraction of lipids from cells and their separation by TLC were performed as described previously [39]. The radioactive lipids on TLC plates were visualized using a Typhoon FLA 7000 (GE Healthcare).

### Others

FACS analysis using Alexa-555 Stx1 B subunits, lysate preparation (RIPA buffer), and Western blot analysis were performed as described previously [39].

## Results

### Gene disruption of *CERT* in HeLa cells by TALENs

We used the “Golden Gate TALEN and TAL Effector Kit (original version)” to construct custom TALEN plasmids [47]. Based on previous reports that the truncation of TALE domains enhanced genome-editing efficiency [41], [42], we modified the TALE scaffold in the original plasmid (pTAL3) to construct pTAL-ModA and -ModB plasmids (Figures 2 and S1). Both plasmids contain truncated N-terminal (+141) and C-terminal (+63) TALE domains (ΔNΔC), the design of which was almost the same as the NΔ152/C+63 TALE truncation variant reported in Miller et al. [41]. pTAL-ModA also contains a set of mutations in the FokI cleavage domain (*Sharkey*), which was reported to improve DNA cleavage activity in zinc finger nucleases [44]. Several reports demonstrated that the combination of truncation in the TALE domain and incubation at 30°C after transfection greatly enhanced genome-editing effeciency [41], [45], [51]; therefore, incubation of cells at 30°C was also employed in this study.

First we attempted to disrupt the *CERT* gene in a HeLa cell line, HeLa-mCAT#8 [39]. The HeLa cell lineage is a pseudo-triploid type and most genes of HeLa cells have 3 or more alleles [35], [36], which require triallelic disruption to achieve complete loss-of-function of the target gene. We selected two TALEN target sites in the PH domain of CERT (CERTv1 and v2, Figures 3A and S2). The PH domain is critical for the transport of ceramide from the ER to the Golgi apparatus and the subsequent synthesis of SM [16]; therefore, in-frame mutations (e.g. 3-base deletion) may also cause loss of function, which raises the probability that they abolish gene functions. Six TALEN pairs for targeting the human *CERT* gene were prepared, in which two assembled repeat modules (CERTv1 and v2) were combined with three TALEN scaffolds (TAL original, TAL-ModA, and TAL-ModB). Genome-editing efficiency was compared among these constructs in the presence of incubation at 30°C. The proportion of *CERT*-deficient HeLa cells was estimated by a rapid method using lysenin, an SM-specific cytolysin [52]. CERT deficiency renders the cells resistant to lysenin because of the reduction in SM [16]. HeLa-mCAT8 cells were transfected with the six TALEN-CERT pairs, and the cells were treated with lysenin to see their viability (Figure 3B). TALEN-CERT pairs with the original scaffold produced only a limited population (less than 15% in TAL original–CERTv2) of lysenin-resistant variants. In contrast, when TALEN-CERT pairs with ΔNΔC in the scaffold was used, about half of TAL-ModA- or TAL-ModB-CERTv2–treated cells (ModA: 50%, ModB: 64%) became lysenin-resistant. These results demonstrated that ΔNΔC in the TALE domain improved genome-editing efficiency, which is consistent with previous reports, whereas *Sharky* mutations in the FokI cleavage domain did not improve efficiency in TALEN.

We then performed indel analysis of the *CERT* gene in TAL-ModA-CERTv2–treated cells by cloning and Sanger sequencing of genomic PCR amplicons around the target sites. Twelve of 24 sequences were found to be mutated (50% NHEJ) (Figure 3C), consistent with the frequency of the loss-of-function phenotype.

### Isolation of *CERT*-deficient clones

**Figure 4:**
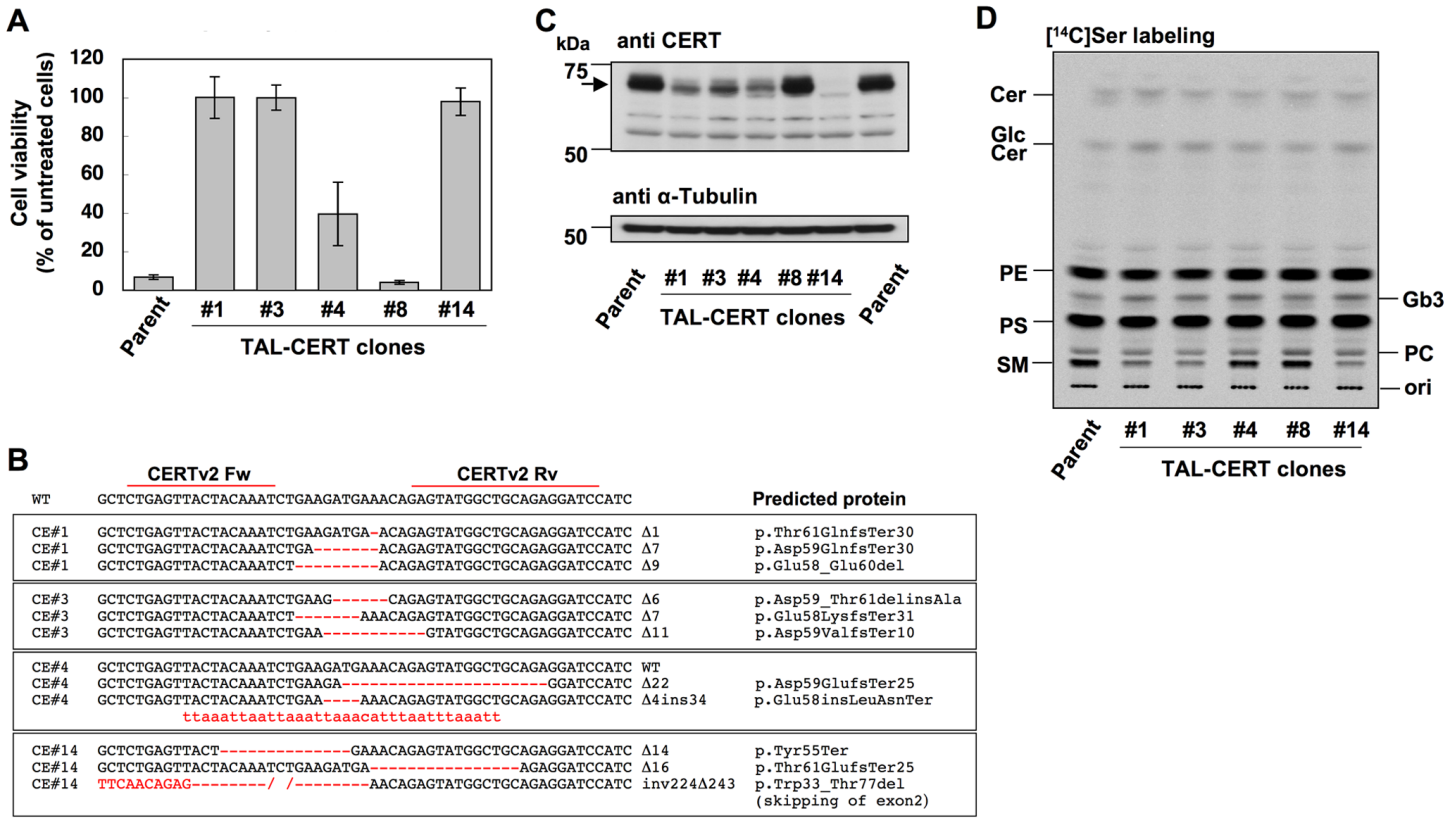
Isolation of *CERT*-deficient HeLa cell clones. A, Resistance to lysenin in HeLa TAL-CERT clones. The clones were treated with lysenin at 670 ng/ml for 2 h. Their viability was estimated by the MTT assay: mean percentage ± S.D. obtained from three independently repeated experiments. B, Indel analysis of *CERT* gene in TAL-CERT clones. Indels are shown in red and their lengths are specified on the right of the sequences. The predicted proteins are indicated based on the recommended description (see Materials and Methods) [50]. C, Protein levels of CERT in TAL-CERT clones. Arrow indicates CERT protein at 68 kD. D, Metabolic labeling of lipids with radioactive serine. TAL-CERT clones were cultured with [^14^C]serine for 16 h, and lipids extracted from the cells were separated by TLC. Radioactive image of an analyzed TLC plate is shown. PE, phosphatidylethanolamine; PS, phosphatidylserine; PC, phosphatidylcholine.

Next, TAL-ModA-CERTv2–treated cells were cloned by limiting dilution. Fourteen clones were purified and their sensitivity to lysenin was examined. Six of 14 clones were lysenin resistant and one was partially resistant (data not shown). The cell clones that grew stably were further propagated and used for further experiments. Clone 1, 3, and 14 (TAL-CE#1, #3, and #14) were completely lysenin resistant and clone 4 (TAL-CE#4) was partially lysenin resistant, while clone 8 (TAL-CE#8) was lysenin sensitive as the parent cell line (Figure 4A).

Indel analysis was then performed on these clones (Figure 4B). In TAL-CE#1 and #3, two of three *CERT* gene alleles had frameshift-causing deletions and one had an in-frame deletion, which coded a deletion of three amino acids in TAL-CE#1 and two amino acids in TAL-CE#3. In TAL-CE#14, two of three *CERT* gene alleles had frameshift-causing deletions and one had a large deletion (243 bp) and inversion (224 bp) (Figure S3A). The large deletion resulted in loss of the splicing acceptor on the 5′ side of exon 2, which suggested that it caused missplicing. Consistently, TAL-CE#14 produced *CERT* mRNA species that were shorter than those of the wild type, and sequence analysis demonstrated that the truncation resulted in the skipping of exon 2 (Figure S3B). The exon-skipped mRNA remained in-frame (45 amino-acid deletion), which caused the deletion of more than half of the PH domain. TAL-CE#4 was a biallelic mutant, and one wild-type allele of the *CERT* gene remained, which was consistent with the phenotype of partial lysenin resistance. TAL-CE#8 had all wild-type *CERT* alleles. Expression levels of CERT protein in these cell clones were examined by Western blot analysis. CERT was detected in TAL-CE#1, #3, and #4 cell clones, although the expression level was lower in these three clones than in the parent cell line and TAL-CE#8 (Figure 4C), it being consistent that one-third of *CERT* alleles was frame–maintained in these clones (Figure 4B). On the other hand, CERT was not observed in TAL-CE#14, although the clone should express a truncated CERT with the deletion of 45 amino acids, the size of which was deduced to be less than 63 kDa compared with 68 kDa in the wild type. Although the reason for this discrepancy is unknown, rapid degradation of the deletion mutant proteins might occur.

*De novo* SM biosynthesis was then investigated in these clones by metabolic labeling with [^14^C]serine (Figure 4D). TAL-CE#1, #3, and #14 showed marked reduction in SM synthesis, whereas TAL-CE#4 showed less reduction in SM than TAL-CE#1, #3, and #14 because TAL-CE#4 still contained one wild-type allele. These results were also consistent with lysenin resistance and supported that the deletion of two or three amino acids in TAL-CE#1 and #3 inactivated the function of the PH domain. The labeled SM exhibited two bands in the thin layer chromatogram (Figure S4), and it is most likely that the upper band mainly represents SM subspecies having a C24:1 acyl chain (C24:1 SM) and the lower band represents SM subspecies having a C16:0 acyl chain (C16:0 SM) from previous reports [53], [54]. Disruption of *CERT* alleles resulted in reduction of both bands (Figure S4). This suggests that CERT can effectively transfer C24:1 ceramide for the synthesis of SM in HeLa cells. On the other hand, the synthesis of GlcCer and Gb3 was not reduced in these TAL-CE#1, #3, and #14, being consistent with our model that CERT primarily contributes to the synthesis of SM, not GlcCer [16]. These clones showed almost no difference in metabolic labeling of phosphatidylserine (PS) and phosphatidylethanolamine (PE) (Figure 4D), ruling out the minor possibility that TALEN treatment caused non-specific perturbations in lipid metabolism.

### Isolation of *UGCG*-deficient clones

**Figure 5:**
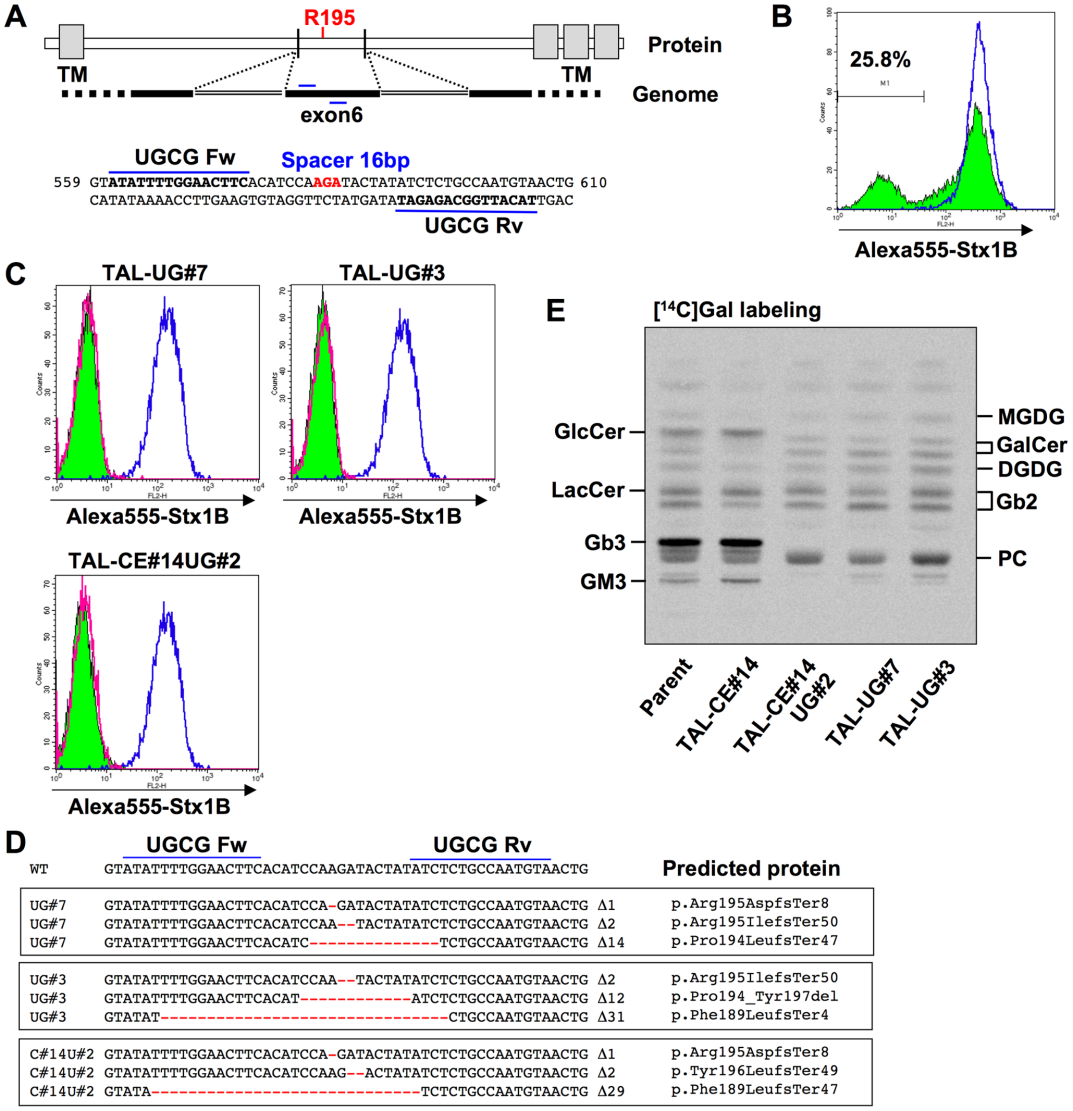
Isolation of *UGCG*-deficient and *CERT/UGCG* double-deficient clones. A, Target sites of TALEN-UGCG pair (TAL-ModA-UGCG) in human *UGCG* gene. The sequences are located in exon 6, which contains the codon of the 195th arginine (R195) essential for the activity. The target sites are shown in bold and the codon of R195 is shown in red. The numbers on the right and left of the sequence indicate the sequence numbers from the A of the translation initiation codon, based on *UGCG* mRNA (accession number D50840). B, Surface expression of StxRs on TALEN-UGCG–treated HeLa cells. HeLa cells were treated with TALEN-UGCG (colored histogram with black line) or empty vectors (blue line), and the cells were stained with Alexa-555-Stx1B. C, Surface expression of StxRs on TAL-UGCG clones (TAL-UG#7 and #3) and a TAL-CERT/UGCG clone (TAL-CE#14UG#2). The clones were stained with Alexa-555-Stx1 B (colored histogram with black line) or not (magenta line) and HeLa-mCAT#8 cells were stained with Alexa-555-Stx1 B (blue line). D, Indel analysis of *UGCG* gene in TAL-UGCG (TAL-UG#7 and #3) and TAL-CE#14UG#2 clones. Deletions are shown in red and their lengths are specified on the right of the sequences. The predicted proteins are indicated based on the recommended description (see Materials and Methods) [50]. E, Metabolic labeling of lipids with radioactive galactose. TAL-UG#7, -UG#3 and -CE#14UG#2 cells were labeled with [^14^C]galactose for 16 h, and lipids extracted from the cells were separated by HPTLC. Radioactive image of an analyzed TLC plate is shown. MGDG, monogalactosyl diacylglycerol; DGDG, digalactosyl diacylglycerol (Galα1-4GalDG); PC, phosphatidylcholine.

GlcCer is a key intermediate in the biosynthesis of complex glycosphingolipids and is synthesized by UDP-glucose:ceramide glucosyltransferase (gene symbol *UGCG*, encoding GlcCer synthase) [21]. We then attempted to generate *UGCG*-deficient HeLa cell lines by TALENs. UGCG has an active site on the cytosol face and a previous report demonstrated some critical amino acids, including the 195th arginine (R195), for its activity [55]. We chose a targeting site of a TALEN pair around the codon of R195 on exon 6, which is expected to destroy the activity even when the TALEN-induced mutation is not frameshifted (Figure 5A). The appearance frequency of *UGCG*-deficient cells was first estimated by using a fluorescent Shiga toxin B-subunit (Alexa 555-Stx1B) that binds to the glycolipid Gb3, a downstream metabolite of GlcCer. About 30% of TALEN-treated cells were Stx1B-binding negative (Figure 5B), suggesting that these cells were *UGCG* deficient. In order to concentrate *UGCG*-deficient cells, TALEN-treated cells were treated with Shiga toxin 1 holotoxin (Stx1), which resulted in elimination of Gb3-positive cells, and then tolerant cells were cloned by limiting dilution. Two clones (TAL-UG#7 and #3) were selected as Stx1B-binding negative clones (Figure 5C). Indel analysis showed that all three alleles of *UGCG* have frameshift-causing deletions in TAL-UG#7, whereas two of three *UGCG* alleles have frameshift-causing deletions and one has an in-frame deletion in TAL-UG#3 (Figure 5D). *De novo* synthesis of glycolipids was examined by metabolic labeling with [^14^C]galactose. Neither GlcCer nor Gb3 was discernibly labeled in either clone (Figure 5E), which confirmed the successful construction of *UGCG*-deficient cell lines. An allele of *UGCG* in TAL-UG#3 codes a mutant protein lacking 4 amino acids containing R195, suggesting that the deletion completely disrupts the function of UGCG. On the other hand, glycolipids synthesized by galactosylceramide (GalCer) synthase and the downstream metabolites, including GalCer, galabiosylceramide (Gb2), monogalactosyl diacylglycerol (MGDG), and digalactosyl diacylglycerol (DGDG), were still observed in *UGCG*-deficient cell lines.

### Isolation of a *UGCG/CERT* double-deficient clone

Ceramide is the common substrate of CERT and UGCG, which suggests that both proteins modulate the amount of ceramide as well as the synthesis of the major membrane sphingolipids, SM and complex glycolipids [56]. However, the effects of *CERT/UGCG* double disruption on cellular functions have not been examined as far as we know. To generate a *CERT/UGCG* double-deficient cell line, TAL-CE#14 cells were treated with the TALEN-UGCG pair as described above, and an Stx1B-binding negative clone (TAL-CE#14UG#2) was isolated (Figure 5C). Indel analysis showed that all three alleles of *UGCG* have frameshift-causing deletions (Figure 5D), and metabolic labeling analysis showed that GlcCer-derived glycolipids including Gb3 are completely lost in the cells as in TAL–UG#7 and #3 cells (Figure 5E). TAL–CE#14UG#2 cells still showed the reduction in SM biosynthesis (Figure S4).

### Isolation of a *B4GalT5*-deficient clone

**Figure 6:**
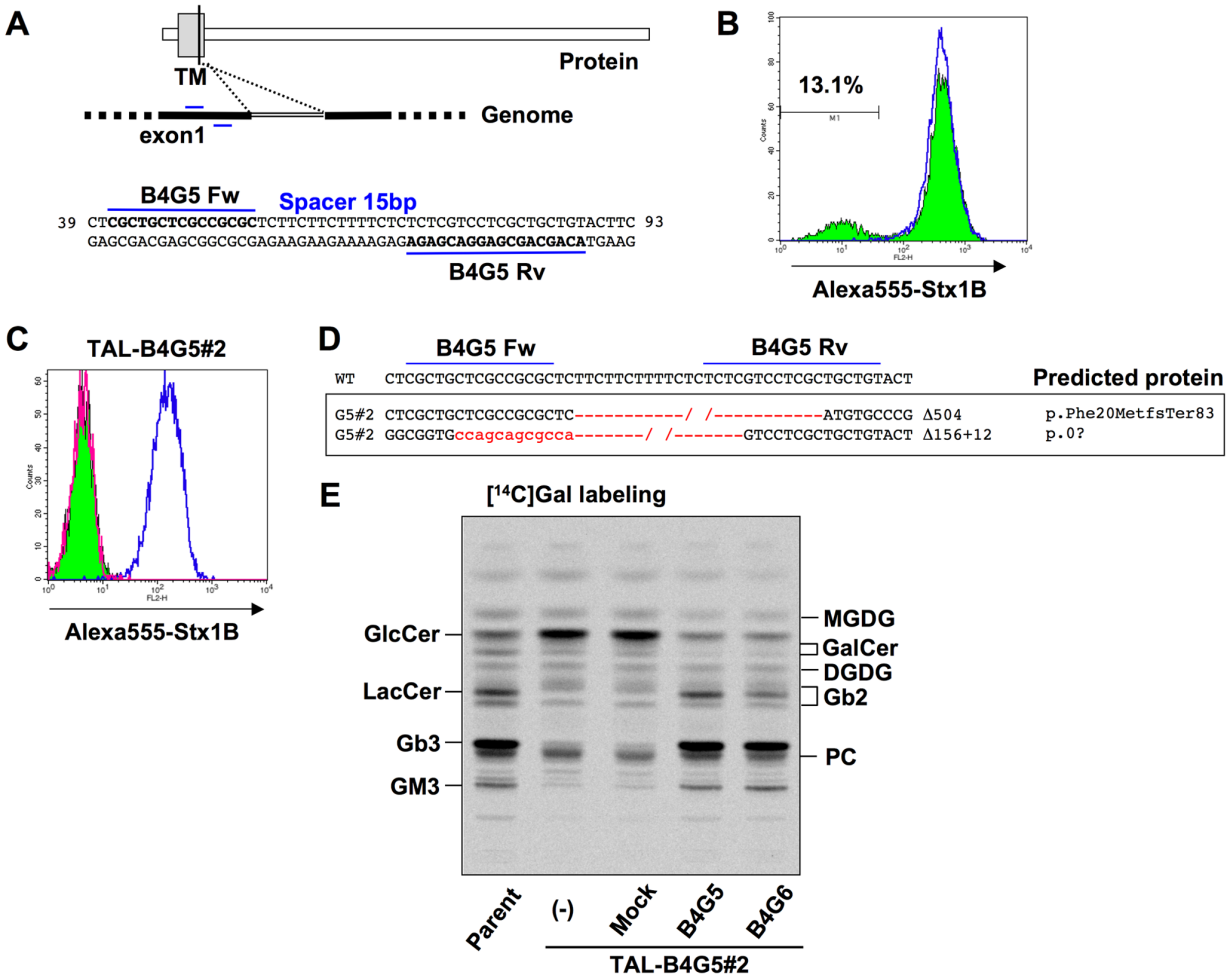
Isolation of a *B4GalT5*-deficient clone. A, Target sites of TALEN-B4G5 pair (TAL-ModA-B4GalT5) in human *B4GalT5* gene. The sequences are located in exon 1, which codes part of the transmembrane domain. The target sites are shown in bold. The numbers on the right and left of the sequence indicate the sequence numbers from the A of the translation initiation codon, based on *B4GalT5* mRNA (accession number AB004550). B, Surface expression of StxRs on TALEN-B4GalT5–treated HeLa cells. HeLa cells were treated with TALEN-B4GalT5 (colored histogram with black line) or empty vectors (blue line), and the cells were stained with Alexa-555-Stx1B. C, Surface expression of StxRs on a TAL-B4GalT5 clone (TAL-B4G5#2). TAL-B4G5#2 cells were stained with Alexa-555-Stx1 B (colored histogram with black line) or not (magenta line) and HeLa-mCAT#8 cells were stained with Alexa-555-Stx1 B (blue line). D, Indel analysis of *B4GalT5* gene in TAL-B4G5 clone (TAL-B4G5#2). The deletion is shown in red and its length specified on the right of the sequence. The predicted proteins are indicated based on the recommended description (see Materials and Methods) [50]. E, Metabolic labeling of lipids with radioactive galactose. TAL-B4G5#2 clone and B4GalT5- or B4GalT6-restored TAL-B4G5#2 cells obtained by retroviral vector–mediated overexpression were labeled with [^14^C]galactose for 16 h, and lipids extracted from the cells were separated by HPTLC. Radioactive image of an analyzed TLC plate is shown.

LacCer is synthesized from GlcCer and UDP-galactose. Previous studies with knockout mice showed that B4GalT5 rather than B4GalT6 is a major LacCer synthase [25]–[27]. To address if this is also true in human cultured cells, we decided to disrupt *B4GalT5* of the HeLa-mCAT#8 cell line by TALENs (Figure 6A). TALEN-B4GalT5 (B4G5)–treated cells were first stained with Alexa 555-Stx1B to see the surface expression of Gb3. About 13% of cells were negative for Stx1B binding, suggesting that B4GalT5 acts as the major LacCer synthase in HeLa cells and *B4GalT5* gene was probably disrupted in this population (Figure 6B). After treatment with Stx1 to concentrate the deficient cells, an Stx1B-binding negative clone (TAL-B4G5#2) was isolated (Figure 6C). Genomic PCR analysis showed that two small bands of *B4GalT5* were detected in TAL-B4G5#2, and sequence analysis verified two truncated *B4GalT5* genome sequences (Figures 6D, S5A and B). One of the sequences contained a 504-bp deletion including the exon 1–intron 1 junction, and the other contained a 128-bp deletion including the translation initiation codon with a 12-bp insertion. Recent reports demonstrated that the copy number around chromosome 20q13.13, at which B4GalT5 is located, in HeLa cell lines was three or four [35], [36], and the reason for seeing only two mutations in TAL-B4G5#2 clone remains to be solved, although no wild-type sequence was detected in our analysis. We instead examined B4GalT5 mRNA in TAL-B4G5#2 to see if there were any transcripts. RT-PCR analysis showed that a set of primers surrounding the start and stop codons of B4GalT5 hardly amplified *B4GalT5* cDNA in TAL-B4G5#2 (Figure S5C). However, a very faint band around 2 kbp could be cloned, and sequence analysis showed that the amplified band contained 3′-truncated exon 1 followed by 5′ and 3′-truncated intron 1, which was connected to exon 2 (Figure S5A). The result indicated that the genomic 504-bp deletion eliminates the splicing donor between exon 1 and intron 1, and a cryptic splicing donor site within intron 1 was instead used to connect to the 5′ side of exon 2. The transcript did not code for a functional protein (Figure S5A). These results suggested that TAL-B4G5#2 lost the functional B4GalT5. *De novo* synthesis of glycolipids in TAL-B4G5#2 showed that only a little Gb3 and GM3 was observed (Figure 6E). Furthermore, labeled LacCer was not observed and instead, GlcCer was accumulated, suggesting that TAL-B4G5#2 lost most LacCer synthase activity. Retroviral overexpression of wild-type *B4GalT5* cDNA in TAL-B4G5#2 restored Stx sensitivity and glycolipid composition in the cells, which confirmed that the deleted mutation of *B4GalT5* is the cause of glycolipid deficiency in TAL-B4G5#2 (Figures 6E, S5D and E). *B4GalT6* cDNA was originally isolated as a LacCer synthase–coding gene [57], and overexpression of *B4GalT6* cDNA in TAL-B4G5#2 considerably rescued the deficiency of LacCer and its metabolites (Figures 6E, S5D and E). Thus, B4GalT5 was likely the major LacCer synthase in HeLa cells but B4GalT6 could also show LacCer synthase activity in cells to a lesser extent.

## Discussion

In this study, we succeeded in creating loss-of-function mutants for *CERT*, *UGCG*, and *B4GalT5*, and also a *CERT/UGCG* double-deficient mutant in a HeLa cell line by TALEN technology. We confirmed that the truncation in the TALE domain with hypothermic incubation enhanced genome-editing efficiency, as already reported [41], [51]. TALEN can choose its target site in a wide range of sequences, which is an important feature when choosing a specific sequence as a TALEN target site. If an amino acid critical for the activity of an enzyme is selected as a target site of TALEN, even an in-frame mutation might cause loss of function. To disrupt the *UGCG* gene, we designed a TALEN pair that was predicted to cleave the codon of R195, which was essential for GlcCer synthase activity shown previously [55]. TAL-UG#3, one of the isolated clones, still contained one allele with an in-frame mutation; however, GlcCer synthase activity was completely lost, probably because the codon of R195 was lost (Figure 5E). Thus, choice of an enzyme active site as a TALEN target site might be effective to raise the probability of isolating loss-of-function mutants, especially in polyploid cell lines, although it depends on the purpose of the experiments. We also chose the PH domain as the TALEN target site of CERT because it is critical for the transport of ceramide from the ER to the Golgi apparatus and the synthesis of SM [16]. TAL-CE#1 and 3, isolated in this study, contained one allele with an in-frame mutation of *CERT*, respectively. If these mutations had not affected the ER-to-Golgi ceramide trafficking, these clones would have shown the same phenotype as TAL-CE#4, which still contained one wild-type allele of the *CERT* gene and showed moderate SM synthesis, as shown in Figure 4D. However, the synthesis of SM in TAL-CE#1 and 3 was almost the same as that in TAL-CE#14, which lost discernible expression of CERT, suggesting that these in-frame mutations in TAL-CE#1 and 3 disrupt the function of the PH domain.

Owing to the new mutant clones of HeLa cells with disruption of sphingolipid-related genes, we could confirm and extend the suggestions/conclusions of several previous studies. Since the discovery of CERT, we have argued that CERT plays a major role in the delivery of ceramide to the synthesis site of SM but not of GlcCer [58], [59], [16]. This argument was mainly based on the results obtained from analysis using the CHO mutant LY-A cell line, in which the endogenous *CERT* gene has a misssense mutation in the PH domain [16]. However, there remained the possibility that CERT-mediated delivery of ceramide to the synthesis site of GlcCer might not depend on its PH domain, thereby exhibiting only a marginal effect on GlcCer synthesis in LY-A cells. This possibility was here rejected by the present study showing that a *CERT*-deficient HeLa cell line, HeLa TAL-CE#14, is also defective in *de novo* synthesis of SM, not GlcCer.

Our previous study indicated that the activity of CERT to transfer ceramide is lower for C24:1 ceramide species than for C16:0 ceramide in a cell-free assay system [60]. However, TLC analysis of *CERT*-deficient HeLa cells in the present study suggests that CERT plays a crucial role in the synthesis of C24:1 SM as well as C16:0 SM.

The present study also demonstrated that B4GalT5 is the major LacCer synthase in HeLa cells because the Stx-binding negative population appeared in the transfection of a pair of TALEN-B4GalT5 plasmids, and TAL-G5#2 clone showed considerable reduction in Gb3 and GM3, both downstream metabolites of LacCer. This result is consistent with previous studies using *B4GalT5*-deficient mice [25]–[27]. *B4GalT6* cDNA was originally isolated as a LacCer synthase–coding gene [57]; however, knockout mice showed no apparent phenotype and it was still obscure to what extent B4GalT6 functions as a LacCer synthase in cells [27]. In this study, we confirmed that B4GalT6 also exhibits discernible activity of LacCer synthase in cells.

These newly established sphingolipid-deficient HeLa mutant clones provide a ‘sphingolipid-modified HeLa cell panel’ together with our previously established stable transfectants (Table 1) [39]. Each cell line contains different sphingolipid compositions against the same genomic background (HeLa-mCAT#8 cells). This cell panel would be useful to elucidate the respective functions of different sphingolipid species including analyses of various types of lipid microdomains and their effects on receptor-mediated signaling and endocytosis, as well as the effects of ceramide metabolism on drug sensitivity.

**Table 1:** A panel of sphingolipid-modified HeLa cell lines.

Cell Lines	Major glycolipid		References
Parent (HeLa-mCAT8)	Gb3		Ref. [39]
HeLa-TAL-UG#7	(-) (GalCer, Gb2)		This study
HeLa-TAL-B4G5#2	GlcCer		This study
HeLa-pLib-ST3Gal5	GM3		Ref. [39]
HeLa-shA4GalT	LacCer		Ref. [39]
HeLa-TAL-CE#14	Gb3	SM ↓	This study
HeLa-TAL-CE#14UG#2	(-) (GalCer, Gb2)	SM ↓	This study

## Supporting Information

Figure S1 **Amino acid sequences of original and modified TALENs.** Underlines in the N-terminal TALE (TALE-N) indicate a nuclear localization signal, and a box indicates an HA tag. Two asterisks in the repeat region indicate the repeat variable di-residue (RVD), which decides the nucleotide-binding specificity of each TALE repeat region. Arrows in FokI cleavage domain (*sharkey*) indicate the mutated amino acids.
(TIF)

Figure S2 **Repeat variable di-residue sequences in the TALENs used in this study.**
(TIF)

Figure S3 **Characterization of the large deletion allele of** ***CERT*** **gene in TAL-CE#14 clone.** A, Schematic diagrams indicate the disruption of *CERT* gene in the genome and the resultant exon 2-skipped mRNA in TAL-CE#14 clone. The numbers on the diagrams indicate the sequence numbers from the A of the translation initiation codon, based on *CERT* mRNA (accession number AY453385). Red arrows show the attachment sites of primers (CERT 5′UTR sense and CERT Exon 4 antisense) used in the RT-PCR analysis shown in B. A partial sequence of the exon 2-skipped cDNA is also shown. SD, splicing donor; SA, splicing acceptor; Ex, exon; In, intron. B, RT-PCR analysis of *CERT* mRNA in TAL-CE#14 clone. P indicates parent cells and #14 indicates TAL-CERT #14 clone. Note that a shorter band is observed in the lane of #14.
(TIF)

Figure S4 **Metabolic labeling of lipids with radioactive serine in** ***CERT*** **-deficient and** ***CERT/UGCG*** **double-deficient clones.** The indicated cells were labeled with [^14^C]serine, and the labeled lipids were separated by TLC. PE, phosphatidylethanolamine; PS, phosphatidylserine; PC, phosphatidylcholine.
(TIF)

Figure S5 **Modification of the human** ***B4GalT5*** **gene.** A, Characterization of the large deletion alleles of *B4GalT5* gene in TAL-B4G5#2 clone. The numbers on the schematic diagrams indicate the sequence numbers from the A of the translation initiation codon, based on *B4GalT5* mRNA (accession number AB004550). Red arrows show the attachment sites of primers used in the genomic PCR (B) and RT-PCR (C). Blue lines show the target sites of TALEN-B4GalT5. The sequence of Δ504 loses the splicing donor of intron 1, and the sequence of Δ156+12 loses the translation initiation codon. SD, splicing donor; SA, splicing acceptor; Ex, exon; In, intron. B, PCR analysis of *B4GalT5* gene in the TAL-B4G5#2 clone with various primer combinations. P indicates parent cells and #2 indicates TAL-B4G5#2 clone. The band size in the leftmost lane is about 8 kbp. Only two truncated forms were detected in the TAL-B4G5#2 clone. C, RT-PCR analysis of *B4GalT5* mRNA in the TAL-B4G5#2 clone. B4GalT5 RI-ATG sense and B4GalT5 Hind-END antisense were used as primers. Note that bands are hardly observed in lane #2 in *B4GalT5* cDNA. D, Restoration of Stx1 sensitivity by retroviral overexpression of B4GalT5 and 6 in TAL-B4G5#2. The indicated cells were treated with Stx1 at 100 pg/ml and cultured for 3 days. Their viability was estimated as described by MTT assay: mean percentage ± S.D. obtained from three independently repeated experiments. E, Western blot analysis of HA-tagged B4GalT5 and B4GalT6 proteins expressed in TAL-B4G5#2 cells.
(TIF)

Text S1 **Primer sequences used in this study.**
(DOC)
