# Supplementary figures and images for: Establishment of HeLa Cell Mutants Deficient in Sphingolipid-Related Genes Using TALENs
Source: PLoS One. 2014 Feb 3;9(2):e88124. doi: 10.1371/journal.pone.0088124 (PMC3912166; doi:10.1371/journal.pone.0088124)

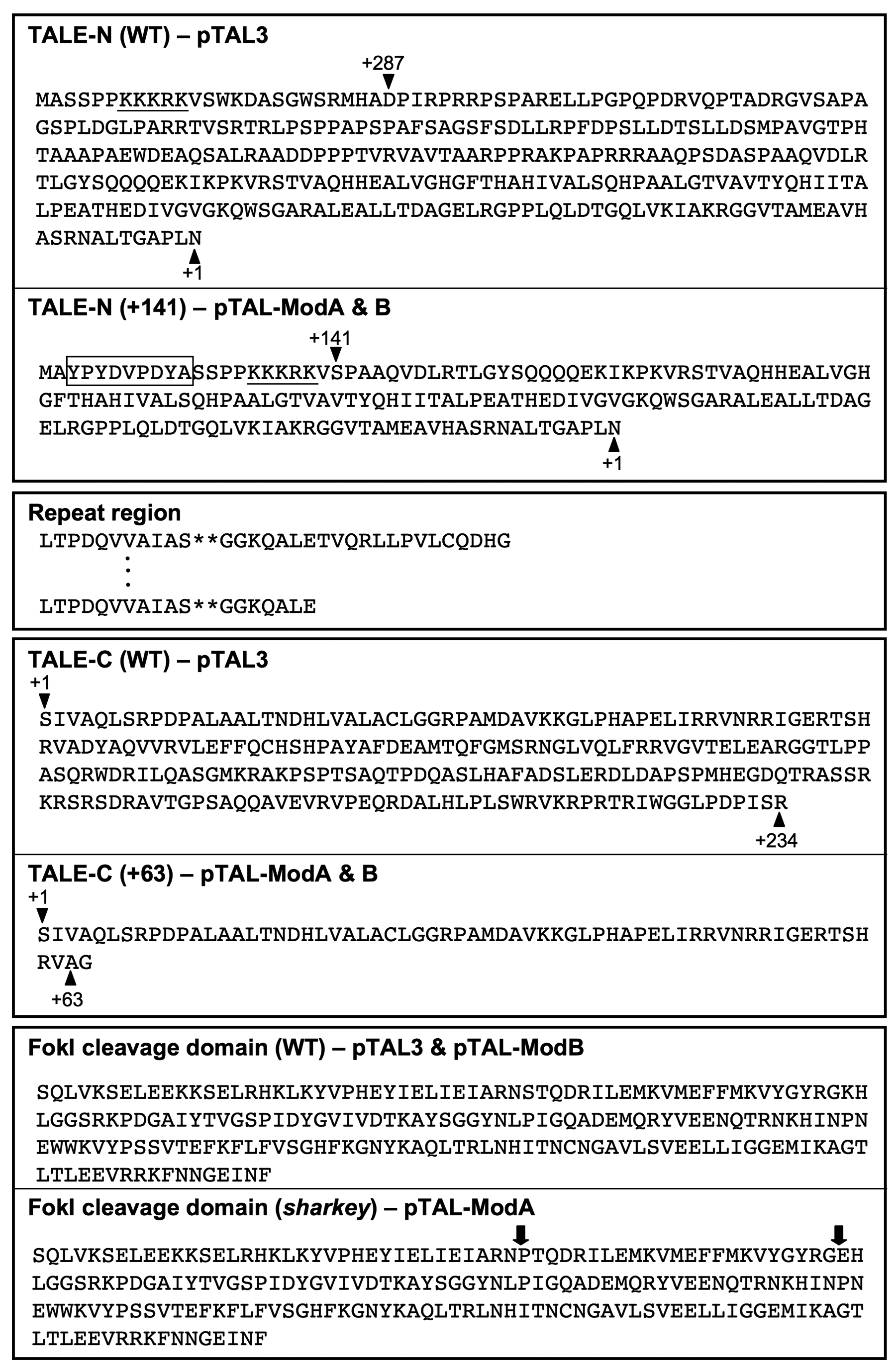

Supplement: Figure S1 — Amino acid sequences of original and modified TALENs. Underlines in the N-terminal TALE (TALE-N) indicate a nuclear localization signal, and a box indicates an HA tag. Two asterisks in the repeat region indicate the repeat variable di-residue (RVD), which decides the nucleotide-binding specificity of each TALE repeat region. Arrows in FokI cleavage domain (sharkey) indicate the mutated amino acids. (TIF) [file pone.0088124.s001.tif]

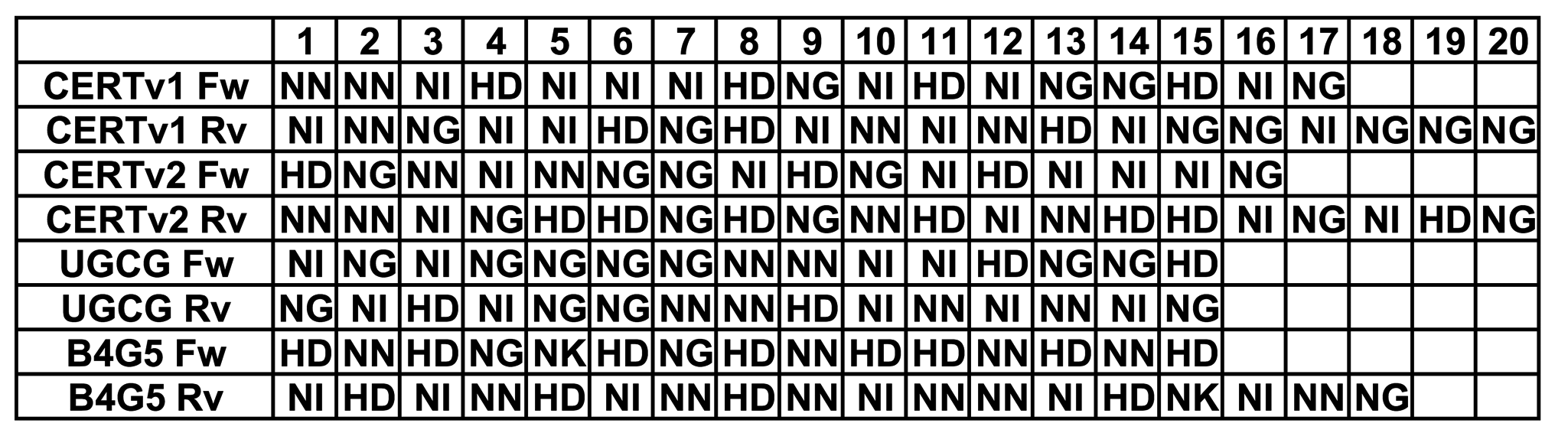

Supplement: Figure S2 — Repeat variable di-residue sequences in the TALENs used in this study. (TIF) [file pone.0088124.s002.tif]

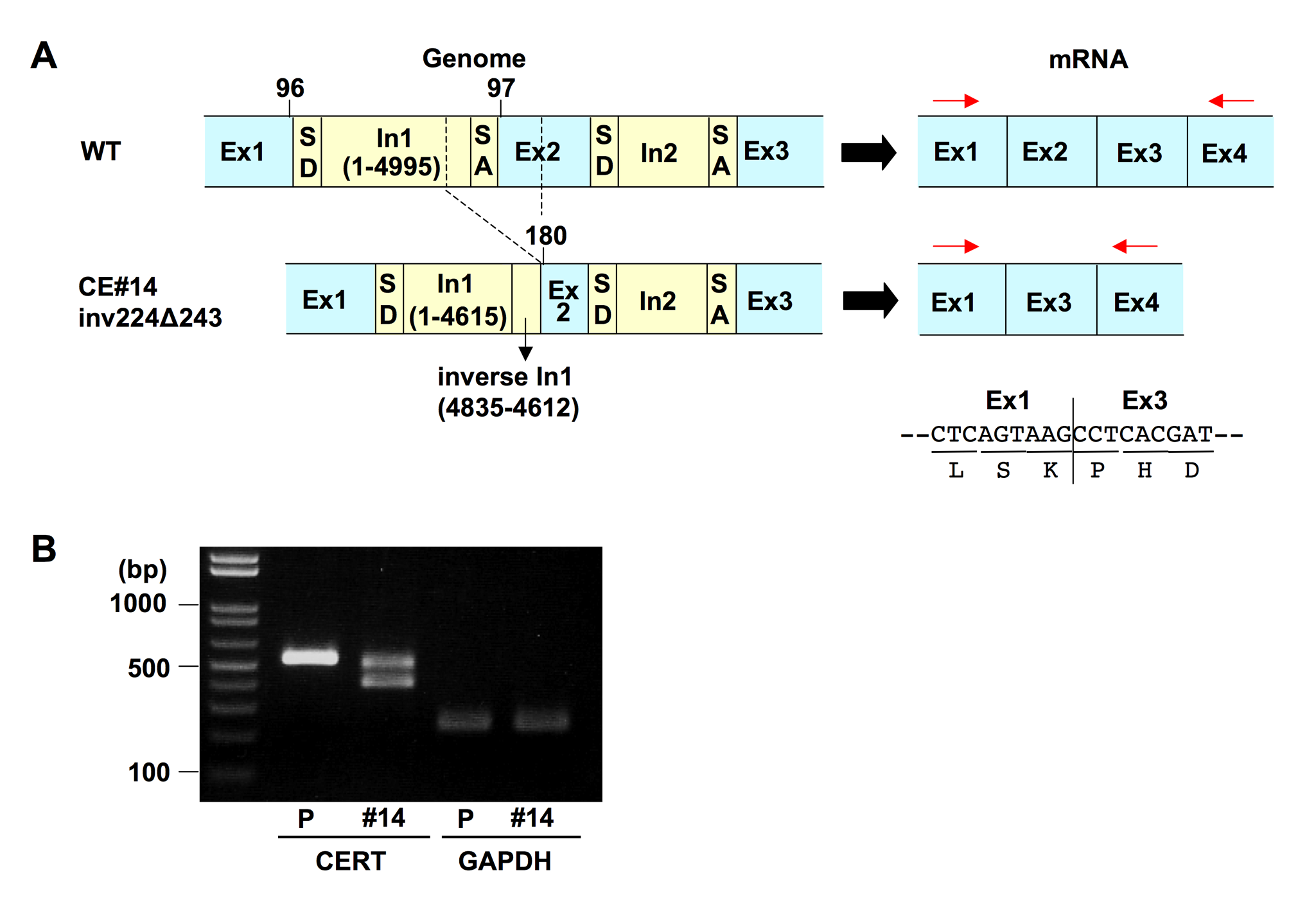

Supplement: Figure S3 — Characterization of the large deletion allele of CERT gene in TAL-CE#14 clone. A, Schematic diagrams indicate the disruption of CERT gene in the genome and the resultant exon 2-skipped mRNA in TAL-CE#14 clone. The numbers on the diagrams indicate the sequence numbers from the A of the translation initiation codon, based on CERT mRNA (accession number AY453385). Red arrows show the attachment sites of primers (CERT 5′UTR sense and CERT Exon 4 antisense) used in the RT-PCR analysis shown in B. A partial sequence of the exon 2-skipped cDNA is also shown. SD, splicing donor; SA, splicing acceptor; Ex, exon; In, intron. B, RT-PCR analysis of CERT mRNA in TAL-CE#14 clone. P indicates parent cells and #14 indicates TAL-CERT #14 clone. Note that a shorter band is observed in the lane of #14. (TIF) [file pone.0088124.s003.tif]

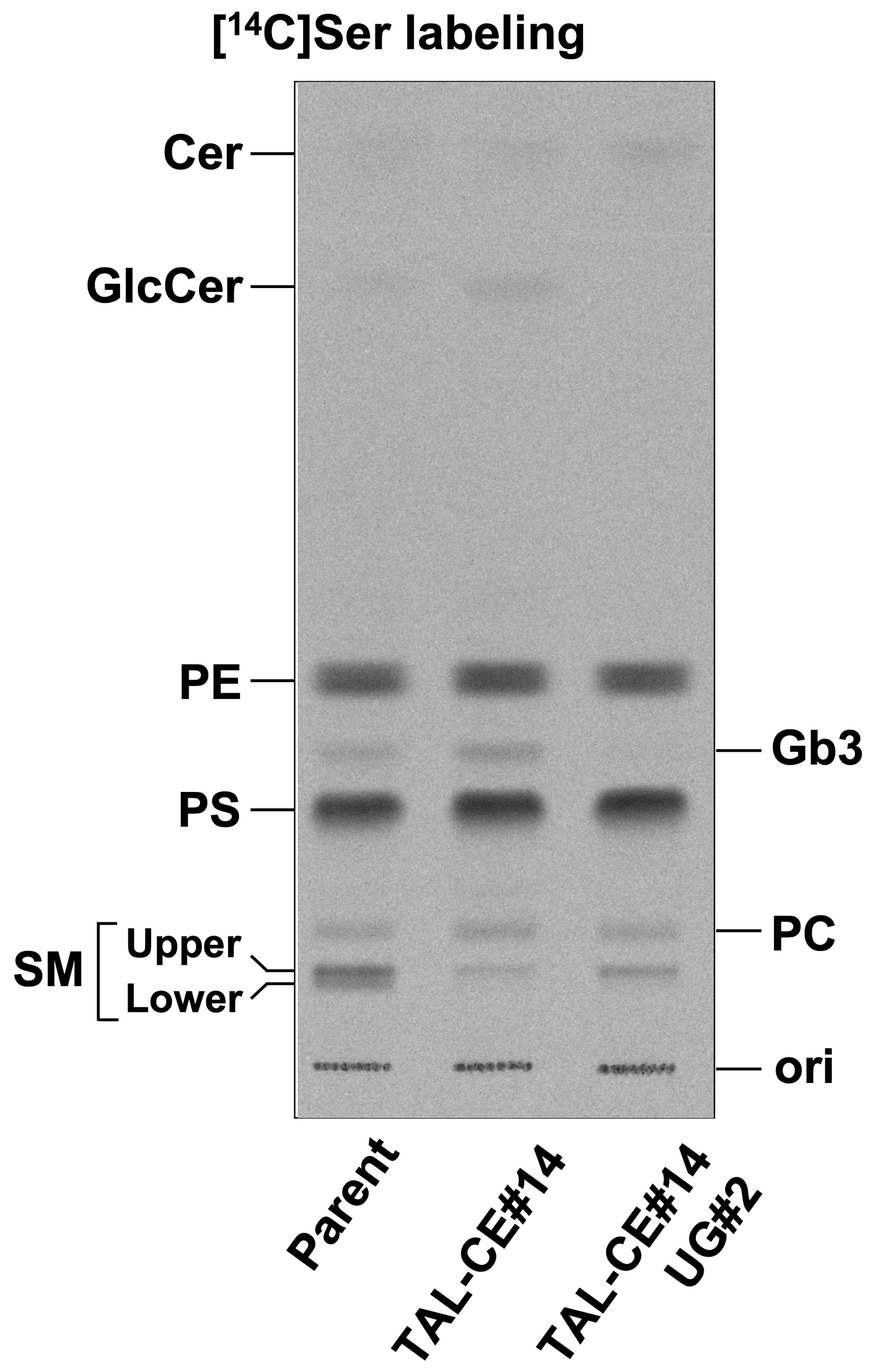

Supplement: Figure S4 — Metabolic labeling of lipids with radioactive serine in CERT -deficient and CERT/UGCG double-deficient clones. The indicated cells were labeled with [14C]serine, and the labeled lipids were separated by TLC. PE, phosphatidylethanolamine; PS, phosphatidylserine; PC, phosphatidylcholine. (TIF) [file pone.0088124.s004.tif]

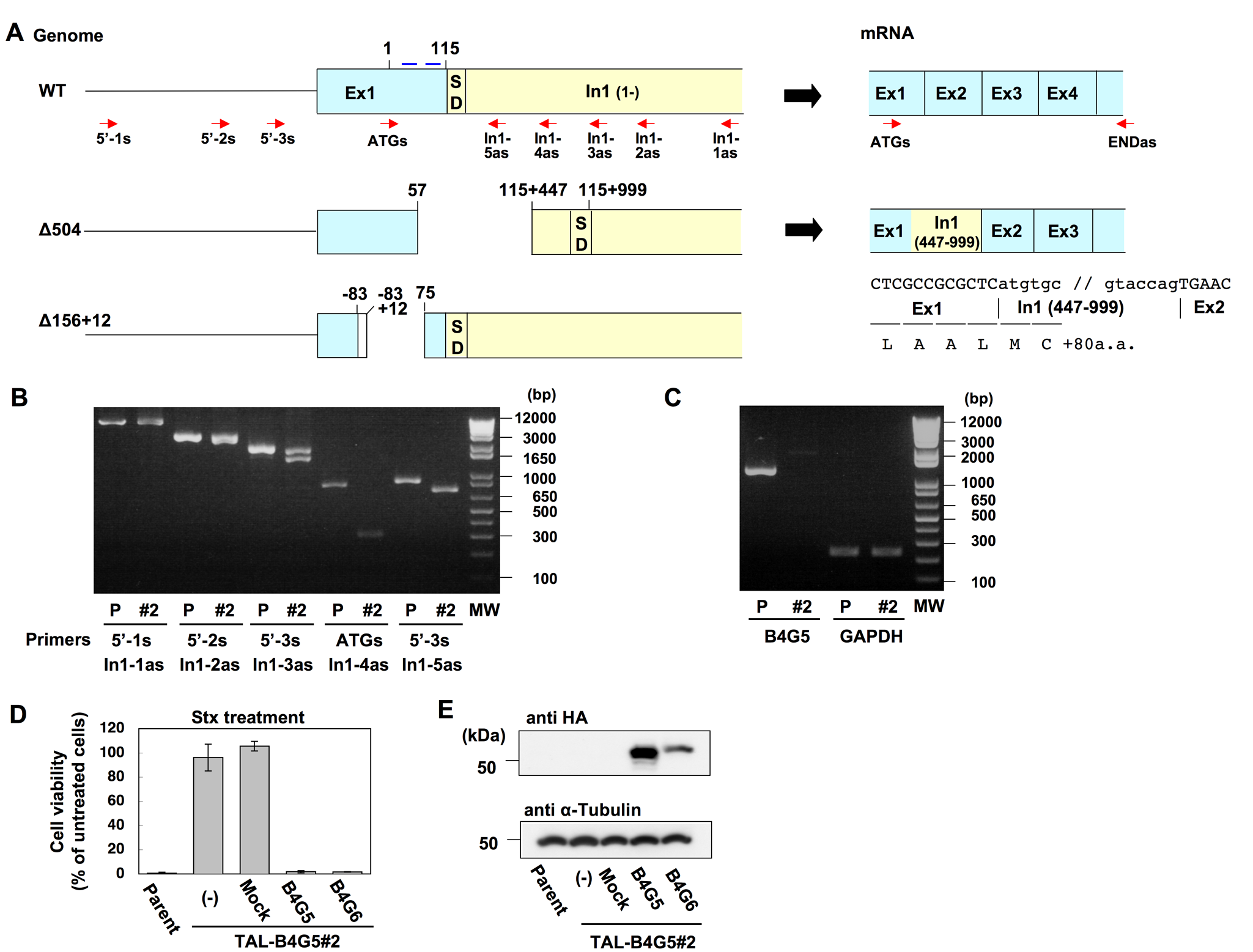

Supplement: Figure S5 — Modification of the human B4GalT5 gene. A, Characterization of the large deletion alleles of B4GalT5 gene in TAL-B4G5#2 clone. The numbers on the schematic diagrams indicate the sequence numbers from the A of the translation initiation codon, based on B4GalT5 mRNA (accession number AB004550). Red arrows show the attachment sites of primers used in the genomic PCR (B) and RT-PCR (C). Blue lines show the target sites of TALEN-B4GalT5. The sequence of Δ504 loses the splicing donor of intron 1, and the sequence of Δ156+12 loses the translation initiation codon. SD, splicing donor; SA, splicing acceptor; Ex, exon; In, intron. B, PCR analysis of B4GalT5 gene in the TAL-B4G5#2 clone with various primer combinations. P indicates parent cells and #2 indicates TAL-B4G5#2 clone. The band size in the leftmost lane is about 8 kbp. Only two truncated forms were detected in the TAL-B4G5#2 clone. C, RT-PCR analysis of B4GalT5 mRNA in the TAL-B4G5#2 clone. B4GalT5 RI-ATG sense and B4GalT5 Hind-END antisense were used as primers. Note that bands are hardly observed in lane #2 in B4GalT5 cDNA. D, Restoration of Stx1 sensitivity by retroviral overexpression of B4GalT5 and 6 in TAL-B4G5#2. The indicated cells were treated with Stx1 at 100 pg/ml and cultured for 3 days. Their viability was estimated as described by MTT assay: mean percentage ± S.D. obtained from three independently repeated experiments. E, Western blot analysis of HA-tagged B4GalT5 and B4GalT6 proteins expressed in TAL-B4G5#2 cells. (TIF) [file pone.0088124.s005.tif]
