# Supplementary material for: Establishment of HeLa Cell Mutants Deficient in Sphingolipid-Related Genes Using TALENs
Source: PLoS One. 2014 Feb 3;9(2):e88124. doi: 10.1371/journal.pone.0088124 (PMC3912166; doi:10.1371/journal.pone.0088124)
Supplement: Text S1 — Primer sequences used in this study. (DOC) [file pone.0088124.s006.doc]

**Used primers**

For deletion of TALE-N and -C scaffolds, attachment of an HA tag, and the mutations on the FokI gene, inverse PCR with self-assembly (PrimeStar Max (Takara)) or seamless cloning (Invitrogen) was performed. Fifteen-base complementary sequences of primers for recombination are underlined.

TAL-deltaC sense (for deletion of TALE-C scaffold)

CGTTGCCGGATCCCAGCTAGTGAAATC

TAL-deltaC antisense (for deletion of TALEvC scaffold)

TGGGATCCGGCAACGCGATGGGACG

TAL-HA sense (for attachment of an HA tag)

CCATATGACGTCCCCGACTACGCTTCCTCCCCTCCAAAGAAAAAG

TAL-HA antisense (for attachment of an HA tag)

GGGGACGTCATATGGGTAGGCCATCTCGAGTTAGATCTAAACTTAG

TAL-deltaN sense (for deletion of TALE-N)

GGTTAGTCCGGCCGCGCAGGTGGATC

TAL-deltaN antisense (for deletion of TALE-N)

GCGGCCGGACTAACCTTTCTCTTTTTC

TAL-SG sense (for a *Sharkey* mutation (S to G))

AAGAAATCCAACTCAGGATAGAATCC

TAL-SG antisense (for a *Sharkey* mutation (S to G))

TGAGTTGGATTTCTTGCGATTTCAATC

TAL-KE sense (for a *Sharkey* mutation (K to E))

TCGTGGTGAACATTTGGGTGGATCAAG

TAL-KE antisense (for a *Sharkey* mutation (K to E))

AAATGTTCACCACGATAACCATAAACC

TAL-602 sense (for sequencing)

GAAAGAAAGCATAGCAATCTAATCTAAG

TAL-1674 antisense (for sequencing)

AACAGTTGCGCAGCCTGAATG

TAL-2006 sense (for sequencing)

CCTAATGAGTGAGCTAACTCAC

TAL-3405 antisense (for sequencing)

GATAATCATCGCAAGACCGG

Primers used for indel analysis, RNA analysis, and cloning (PCR and sequencing) are as follows:

CERT Intron 1-1 sense

GTGATCTTCACTTTGCAGCAAGAC

CERT Intron 1-2 sense

CATGAAGGTACTTAGGAGAGGATG

CERT Intron 2-1 antisense

CCTACAAAGTAACTGTCCCAGC

CERT Intron 2-2 antisense

AATTGCTTGAACCTGGGAGGTC

CERT Exon 2 sense

TGGACAAACTACATTCATGGGTG

CERT Exon 2 antisense

TGTGATGACAGCCTTGCTAAG

CERT EcoRI-UTR-ATG sense

GGTGAATTCGCCTCCATGTCGGATAATCAGAGCTG

CERT Xho-END antisense

GTTACTCGAGGAACAAAATAGGCTTTCCTGC

CERT 5’UTR sense

CTCTTCGCTTCGCCATCC

CERT Exon 4 antisense

GGATGTTGCAGAGTAGCC

UGCG Intron 5 sense

CTAATCATACACGTTTGTGGTTTTTTGGG

UGCG Exon 6 antisense

CTGCTTGATCCAACACATCTTTTCTC

B4GalT5 EcoRI-ATG sense

ACAGAATTCCTGGCTGCAGCATGCGCG

B4GalT5 HindIII-END antisense

ACAAAGCTTGTACTCGTTCACCTGAGC

B4GalT5 5’-1 sense

CAGTCACTGCACACCTGTCGT

B4GalT5 5’-2 sense

CTTGGGTGAATTGCCTTTCCATCTAGA

B4GalT5 5’-3 sense

CGCCAACAACTGTGTCTGGCA

B4GalT5 Intron1-1 antisense

GAGATGGTACCCCATTTCACGGA

B4GalT5 Intron1-2 antisense

CTGCCTGTTCTATCTTCCCAGATCTA

B4GalT5 Intron1-3 antisense

GGCTTAGTAACCTGCCCAAGGT

B4GalT5 Intron1-4 antisense

ATTGAATGGGGGTGGACCTCTG

B4GalT5 Intron1-5 antisense

CAGGACTCCGGAGTCTTCGG

GAPDH sense

GAGTCAACGGATTTGGTCGT

GAPDH antisense

TTGATTTTGGAGGGATCTCG
